# Molecular Mechanisms Mediating a Deficit in Recall of Fear Extinction in Adult Mice Exposed to Cocaine *In Utero*

**DOI:** 10.1371/journal.pone.0084165

**Published:** 2013-12-17

**Authors:** Zeeba D. Kabir, Aaron C. Katzman, Barry E. Kosofsky

**Affiliations:** 1 Division of Pediatric Neurology, Department of Pediatrics, Weill Cornell Medical College, New York, New York, United States of America; 2 Brain and Mind Institute, Weill Cornell Medical College, New York, New York, United States of America; 3 Department of Psychiatry, University of Iowa, Iowa City, Iowa, United States of America; Rutgers University, United States of America

## Abstract

Prenatal cocaine exposure has been shown to alter cognitive processes of exposed individuals, presumed to be a result of long-lasting molecular alterations in the brain. In adult prenatal cocaine exposed (PCOC) mice we have identified a deficit in recall of fear extinction, a behavior that is dependent on the medial prefrontal cortex (mPFC) and the hippocampus. While we observed no change in the constitutive expression of brain derived neurotrophic factor (BDNF) protein and mRNA in the mPFC and hippocampus of adult PCOC mice, we observed blunted BDNF signaling in the mPFC of adult PCOC mice after fear extinction compared to the control animals. Specifically, during the consolidation phase of the extinction memory, we observed a decrease in BDNF protein and it’s phospho-TrkB receptor expression. Interestingly, at this same time point there was a significant increase in total *Bdnf* mRNA levels in the mPFC of PCOC mice as compared with controls. In the *Bdnf* gene, we identified decreased constitutive binding of the transcription factors, MeCP2 and P-CREB at the promoters of *Bdnf* exons I and IV in the mPFC of PCOC mice, that unlike control mice remained unchanged when measured during the behavior. Finally, bilateral infusion of recombinant BDNF protein into the infralimbic subdivision of the mPFC during the consolidation phase of the extinction memory rescued the behavioral deficit in PCOC mice. In conclusion, these findings extend our knowledge of the neurobiologic impact of prenatal cocaine exposure on the mPFC of mice, which may lead to improved clinical recognition and treatment of exposed individuals.

## Introduction

Prenatal exposure to cocaine in both animals and humans has been associated with changes in brain structure [1–5] and cognitive impairments including deficits in attention and learning [6–10]. Utilizing a fear extinction learning paradigm, we recently reported that prenatal cocaine exposed mice, heterozygous for the brain-derived neurotrophic factor (Bdnf) Val66Met allele, exhibit a deficit in recall of an extinguished cue-conditioned fear [10]. Furthermore, we identified decreased mature BDNF protein in the medial prefrontal cortex (mPFC) of prenatal cocaine exposed Val66Met mice during extinction training, suggesting this as a possible mechanism mediating the deficit in fear extinction [10]. Extinction of a cue-conditioned fear requires suppression of a conditioned response elicited by a stimulus that no longer predicts reinforcement. This behavior is a well-established learning paradigm that involves several brain regions including the mPFC and hippocampus [11]. The infralimbic subdivision (IL) of the mPFC is the anatomical region that encodes the extinction memory [12–14] and influences the fear response by directly suppressing firing of the amygdala [15–17], while the hippocampus modulates the contextual information of extinction [15] via its connections with the mPFC and the amygdala [18,19]. BDNF, a key regulator of synaptic plasticity [20,21] has been shown to enhance extinction memory [19]. Specifically, increased BDNF protein within the IL plays an integral role in enabling the recall of the extinction memory [19]. 

The *Bdnf* gene is comprised of eight 5’ exons that get differentially spliced onto the 3’ coding exon (exon IX) [20]. Expression of *Bdnf* exons I and IV are activity-driven in a calcium dependent manner in part mediated by the transcription factors CREB and methyl CpG binding protein 2 (MeCP2) [22–25]. All *Bdnf* transcripts containing the coding exon IX get translated into the pro-form of the protein, which when cleaved generates the functionally mature form of BDNF (mBDNF) [26,27]. At the synapse mBDNF binds the tropomyosine receptor kinase B (TrkB) activating its downstream signaling pathway resulting in increased gene expression contributing to synaptic changes [20,28]. By activating the TrkB receptor (via phosphorylation), enhanced BDNF signaling represents a ‘synaptic tag’ that is required for protein synthesis dependent late-LTP [21]. 

In this study we extend our previous findings in [10] to identify a deficit in recall of an extinguished cue-conditioned fear in adult prenatal cocaine exposed mice that is a result of a distinct molecular pathway, specifically in the mPFC and provide novel findings at the level of the protein, mRNA and the gene. Using a transplacental cocaine paradigm we have mapped the behavioral deficit in fear extinction to decreased molecular plasticity of *Bdnf* in the mPFC of prenatal cocaine exposed adult offspring. Such mechanisms may be of more generalized molecular and behavioral significance regarding the epigenetic origins of behavioral deficits, and may have clinical implications for targeted treatments of affected individuals.

## Materials and Methods

### Animals and prenatal treatments

Adult wild-type male mice (2-3 months old) on the Swiss Webster background were used for all experiments. A transplacental cocaine treatment regimen as previously described [10] was used to expose mouse embryos to cocaine. Adult timed-pregnant Swiss Webster dams purchased from Taconic (Germantown, New York) were allowed access to food and water ad libitum and housed on a 12-hour (7:00AM light-7:00PM dark) cycle. The day of vaginal plug detection was considered as embryonic day 0 (E0) and the day of birth as postnatal day 0 (P0). Each dam was assigned to one of two treatment groups and received twice-daily subcutaneous (SC) injections (at 7:00AM and 7:00PM) from E8 to E17, inclusive, of cocaine HCl (Sigma-Aldrich, St. Louis, Missouri; 20 mg/kg/injection, SC, dissolved in saline) totaling 40 mg/kg per day (offspring referred to as PCOC for prenatal cocaine treated) or 0.9% saline (offspring referred to as PSAL for prenatal saline treated). Though dams injected with cocaine gained less weight during gestation, there was no effect of prenatal cocaine treatment on the number of live born pups per litter (data not shown). Within 24 hours of birth, all pups were surrogate fostered to control dams (Swiss Webster; Taconic Labs), which had delivered within the preceding 24-72 hours. Pups were weaned and group housed (3-5/ cage) by gender on P21. Only male offspring were used for these studies. To avoid the problem of oversampling [29], no more than one animal per litter was used for any of the experiments reported. All experimental protocols were approved by the Weill Cornell Medical College Institutional Animal Care and Use Committee, and were in accordance with NIH directives for animal studies. 

### Cue-induced fear conditioning & extinction

The behavioral paradigm used is the same as that in [10]. The conditioning apparatus consisted of a mouse shock-chamber set up in a sound attenuated box that was scented with peppermint odor (0.1% peppermint). On day 1, the conditioning day, animals were allowed to habituate to the conditioning chamber for 2 minutes after which they received 5 conditioning trials consisting of a 30 second presentation of a (2.9kHz, 84-dB) tone (conditioned stimulus) that co-terminated with a 0.7 mA foot shock (unconditioned stimulus) delivered through the grid floor during the last 1 second of the tone. Between each conditioning trial there was a 30 second inter-trial interval (ITI). Following conditioning, mice were returned to their home cages. On days 2, 3 and 4, the behavior was conducted in a novel chamber (circular in shape, with green walls, and scented with 0.1% lemon odor), where mice were allowed to acclimate for 2 mins, and were then presented with 5 tones (30 sec, 2.9kHz, 84-dB, ITI=30 secs). On days 2 and 3, extinction training was performed while on day 4 the test for extinction recall was performed. On each day, mice were recorded using FreezeFrame and freezing was measured via automated analysis using FreezeView (Coulbourn Instruments, Whitehall, PA). Freezing was expressed as a percentage of the 30 sec tone. On days 1, 2 and 3 freezing during each of the 5 tone presentations is reported. On day 4, the average percent freezing during all of the 5 tones is reported.

### Tissue collection

**Figure 1:**
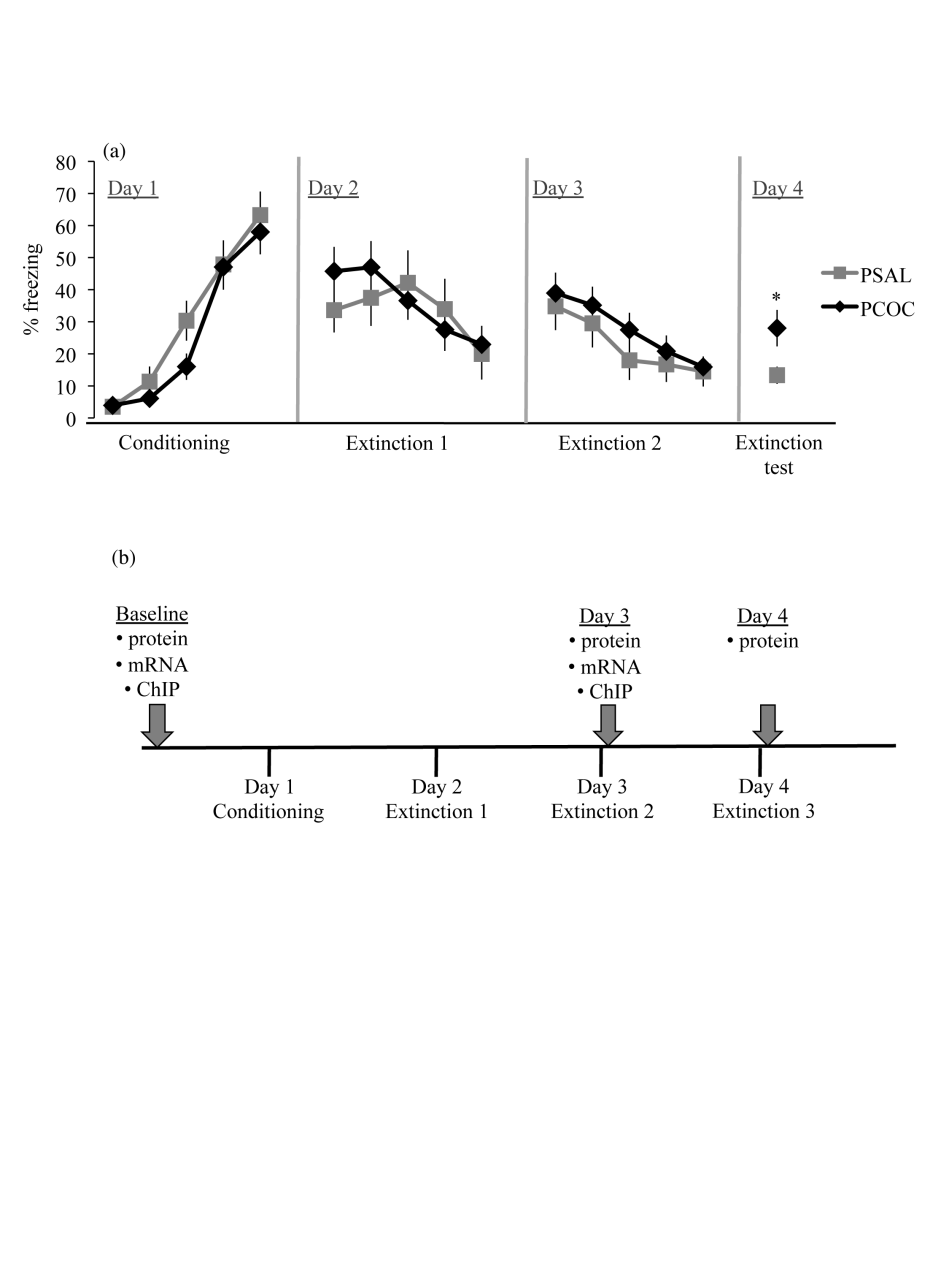
Deficit in recall of an extinguished cue-conditioned fear in prenatal cocaine exposed mice. (**a**) Percent freezing to the tone. On day 1, mice were fear conditioned with five 30 sec tones that co-terminated with a 1 sec 0.7mA shock. On days 2 and 3, mice received extinction training in a novel environment and on day 4 were tested for extinction recall. Both prenatal treatment groups showed significant levels of acquisition on day 1. On days 2 and 3, all animals showed significant within-session extinction with no difference in freezing between PSAL and PCOC mice. At extinction testing on day 4, PCOC mice showed a deficit in recall of fear extinction (*p<0.05). Error bars represent the mean ± SEM (PSAL n=9 from 9 litters; PCOC n=9 from 9 litters). (**b**) Overview of the experimental paradigm. Brain tissue for molecular experiments was collected at three different time points from separate cohorts of animals. For analysis of constitutive levels of MeCP2 and P-CREB binding, Bdnf mRNA and protein expression, and phosphorylated TrkB analyses, tissue was collected in adult PSAL and PCOC animals that were not subjected to any behavioral testing (baseline). For molecular analyses during the consolidation phase of the extinction memory, tissue was collected immediately after extinction training on day 3 (Day 3) or immediately after the test for extinction recall on day 4 (Day 4).

Tissue was collected in separate cohorts of adult mice at three different time points – baseline, immediately after day 3 of the behavior, and immediately after day 4 of the behavior (Figure 1b). Mice were euthanized by rapid decapitation, whole brains were dissected from the skull and the mPFC and hippocampus were dissected. For BDNF protein, tissue from the left hemisphere was used and for mRNA analyses, tissue from the right hemisphere was used. For immunoprecipitation experiments, tissue from both hemispheres was used.

### Cannulae placement

Adult mice were stereotaxically fitted with bilateral guide cannulae targeting the IL subdivision of the mPFC (stereotaxic coordinates according to the atlas of Paxinos & Watson were: anteroposterior, AP, +1.7mm; mediolateral, ML, +2.2mm; dorsoventral, DV, -3.3mm; angled 30° toward the midline in the coronal plane). Adult mice were anesthetized with a xylazine (20 mg/ml) and ketamine (100 mg/ml) cocktail, and mounted to a stereotaxic surgical apparatus (David Kopf Instruments, Tujunga, CA). A midline incision was made atop the scalp, skin was retracted, and the head was leveled based on the horizontal positions of bregma and lambda. Two holes were formed through the skull using a 25-gauge needle and intracranial guide cannulae (Plactics One, Inc., Roanoke, VA) were inserted and glued to the skull. ‘Dummy’ probes (33-gauge) (maintained in position following cannulae placement) were cut flush with the guide cannula (26 gauge) while the injector probes (33 gauge) (positioned prior to BDNF infusion) extended 1 mm below the cannula into the brain. Cannulae placements were verified by dye injections followed by coronal sectioning on the cryostat. Mice were allowed to recover from surgery for 5-7 days before initiation of the behavior. Animals in which the cannulae showed an incorrect placement were removed from the analyses.

### Intracranial drug infusion

Recombinant BDNF was infused into the IL immediately after day 3 of the behavior in awake animals at a dose of 0.75ug/0.5ul/side. This dose when administered in the IL of rats has been shown to enhance extinction memory [19]. BDNF was dissolved in sterile PBS and infused at a rate of 0.25ul/min as has previously been published [19]. As a control sterile PBS (0.5ul) was infused at the same rate.

### BDNF ELISA

Mature BDNF protein level was measured using the BDNF E_max_ ImmunoAssay (ELISA) system (Promega, Madison, WI) with recombinant mature BDNF as a standard as described in [10]. Protein was extracted and quantitated following the manufacturers protocol. Tissue samples were homogenized in lysis buffer (150mM NaCl, 1% Triton X-100, 25mM HEPES, 2mM NaF) containing phosphatase and protease inhibitors and incubated at 4°C on a rotator for 1 hour. The homogenized tissue was centrifuged at maximum speed and the supernatant containing total protein was collected and quantified using the BCA protein assay kit (Thermo Fisher Scientific, Rockford, IL). Each sample was diluted 1:1 with block and sample buffer (BSB) and placed in designated wells of a 96-well plate previously coated with BDNF antibody in carbonate buffer (25mM Na_2_CO_3_ and 25mM Na_2_HCO_3_, pH 9.7, incubated at 4°C) and then blocked with BSB. A second coating of primary anti-human BDNF antibody was added followed by the horseradish peroxidase-conjugated secondary antibody. The colorimetric reaction was initiated by tetramethylbenzidine. After 10 minutes the reaction was stopped with 1N HCl and the absorbance was read at 450 nm on a plate reader (iMark Absorbance Microplate Reader, Bio-Rad Laboratories, Hercules, CA). Standard and samples were performed in duplicates. 

### Phosphorylated TrkB analyses

Fresh tissue was homogenized in IX RIPA buffer (Millipore, Billerica, MA) containing phosphatase and protease inhibitors and incubated at 4°C on a rotator for 1 hour. The homogenized tissue was centrifuged and the supernatant containing total protein was collected and quantified using the BCA protein assay kit (Thermo Fisher Scientific, Rockford, IL). For the immunoprecipitation, 800ug of total protein lysate was incubated overnight with 8ug TrkB antibody (Cat# 07-225, Millipore, Billerica, MA) and hydrated protein A-sepharose beads (Sigma, St. Louis, MO). The immunoprecipitated samples were washed with 1X RIPA buffer and eluted by boiling. The eluted protein was separated on a 10% Bis-Tris gel (Life Technologies, Grand Island, NY) along with a Kaleidoscope-prestained standard (Bio-Rad, Hercules, CA). Blots were incubated in primary antibody (pY99 1:5000 (Cat# sc-7020), TrkB 1:1000 (Cat# sc-8316), Santa Cruz Biotechnology, Santa Cruz, CA; Calnexin 1:200 (Cat# ab22595), Abcam, Cambridge, MA) for 12-16 hours at 4°C. Secondary antibody incubations were performed at room temperature for 1 hour (horseradish peroxidase-linked IgG conjugated horse anti-mouse 1:4000 for pY99, or goat anti-rabbit 1:5000 for TrkB and Calnexin, Vector Laboratories, Burlingame, CA). Membranes were visualized with Western Lightning Chemiluminescence solution (Perkin Elmer Life Science, Boston, MA). Optical density was analyzed using NIH Image (NIH, Bethesda, MD). Calnexin was used to normalize levels of total and phosphorylated TrkB. 

### Real time RT-PCR

Total RNA was extracted and reverse transcribed as described in [30]. For qPCR performed on samples that underwent ChIP, the parameters used were as in [31]. Primers for *Bdnf* exon IV were previously reported in [32] and for *Bdnf* exon I in [33]. Prior to use, all primer sequences were blasted to confirm homology with the mouse *Bdnf* gene. The immunoprecipitated levels were normalized to the total input control.

### Chromatin immunoprecipitation (ChIP)

ChIP assays were performed on fragmented chromatin using the ChIP-IT Express kit (Active Motif, Carlsbad, CA) as per the protocol supplied by the company. For each sample, two ChIP reactions were performed – one for MeCP2 and the other for P-CREB. An aliquot of the fragmented chromatin was set aside as total input control. For the ChIP reaction, 100ul of the fragmented chromatin was mixed with protein G magnetic beads and 5ul of anti-MeCP2 antibody (Cat# ab2828, Abcam, Cambridge, MA) or anti-P-CREB antibody (Cat# 17-10131, Millipore, Billerica, MA) and rotated overnight at 4°C. The beads were washed with wash buffer and the DNA was immunoprecipitated.

### Statistical analyses

Prior to statistical analysis the outlier test was performed, excluding animals with values greater than two standard deviations from the mean. For the behavioral data, a two-way (prenatal treatment X tone) ANOVA for each day was calculated using the percentage freezing during each of the five tones on that day as the dependent variable. When a main effect was found (p<0.05), post-hoc comparisons using the Bonferroni correction were performed for individual tones within a test day. For post-hoc analyses on day 4 the average percent freezing over all 5 tones was analyzed. On day 4 for the behavioral rescue experiment, a two-way (prenatal treatment X BDNF infusion) ANOVA was calculated and when a main effect on the average percent freezing over all 5 tones was found the Bonferroni post-hoc test was performed.

ChIP, qPCR, and ELISA data were analyzed by a two-way (prenatal treatment X day) ANOVA and when significant (p<0.05) post-hoc comparisons using the Bonferroni correction were performed. For total TrkB and phosphorylated TrkB analyses, a Student’s t-test were performed at each individual time point measured.

## Results

### Prenatal cocaine exposure results in a deficit in recall of an extinguished cue-conditioned fear

To study the impact of prenatal cocaine exposure on cognition, a fear extinction behavioral paradigm was utilized. On day 1, PCOC and PSAL animals showed an increase in freezing with each tone-shock pairing (F_4,120_=41.58, p<0.0001) with no main effect of prenatal treatment, indicating that prenatal cocaine exposure did not impact the sensitivity to foot shock. On days 2 and 3, all animals exhibited a decrease in freezing from the first to the fifth tone (day 2: F_4,120_=2.388, p=0.05; day 3: F_4,120_=4.988, p<0.0001) with no difference between PCOC and PSAL mice. On day 4, there was a main effect of freezing during the tones (F_4,120_=2.62, p<0.05) together with a main effect of prenatal treatment (F_1,120_=20.4, p<0.0001). Post-hoc analysis revealed a significant increase in freezing in PCOC mice compared to PSAL mice (Figure 1a). While the PSAL animals had extinguished the learned fear, the PCOC mice continued to display increased freezing to the cue suggesting that prenatal cocaine exposure results in a deficit in recall of an extinguished cue-conditioned fear without impacting the acquisition of the conditioned fear.

### Prenatal cocaine exposure results in decreased mature BDNF and phosphorylated TrkB protein levels in the mPFC during consolidation of the extinction memory

To identify a molecular mechanism that may contribute to the behavioral deficit observed in adult PCOC mice, we focused our studies on *Bdnf* in the mPFC and hippocampus. As we observed the deficit in fear extinction in adult PCOC mice on day 4 of the behavioral paradigm we hypothesized that the deficit was due to a deficiency in the consolidation of the extinction memory between day 3 to day 4. Hence we examined changes in *Bdnf* during the consolidation phase of the extinction memory (Figure 1b). 

**Figure 2:**
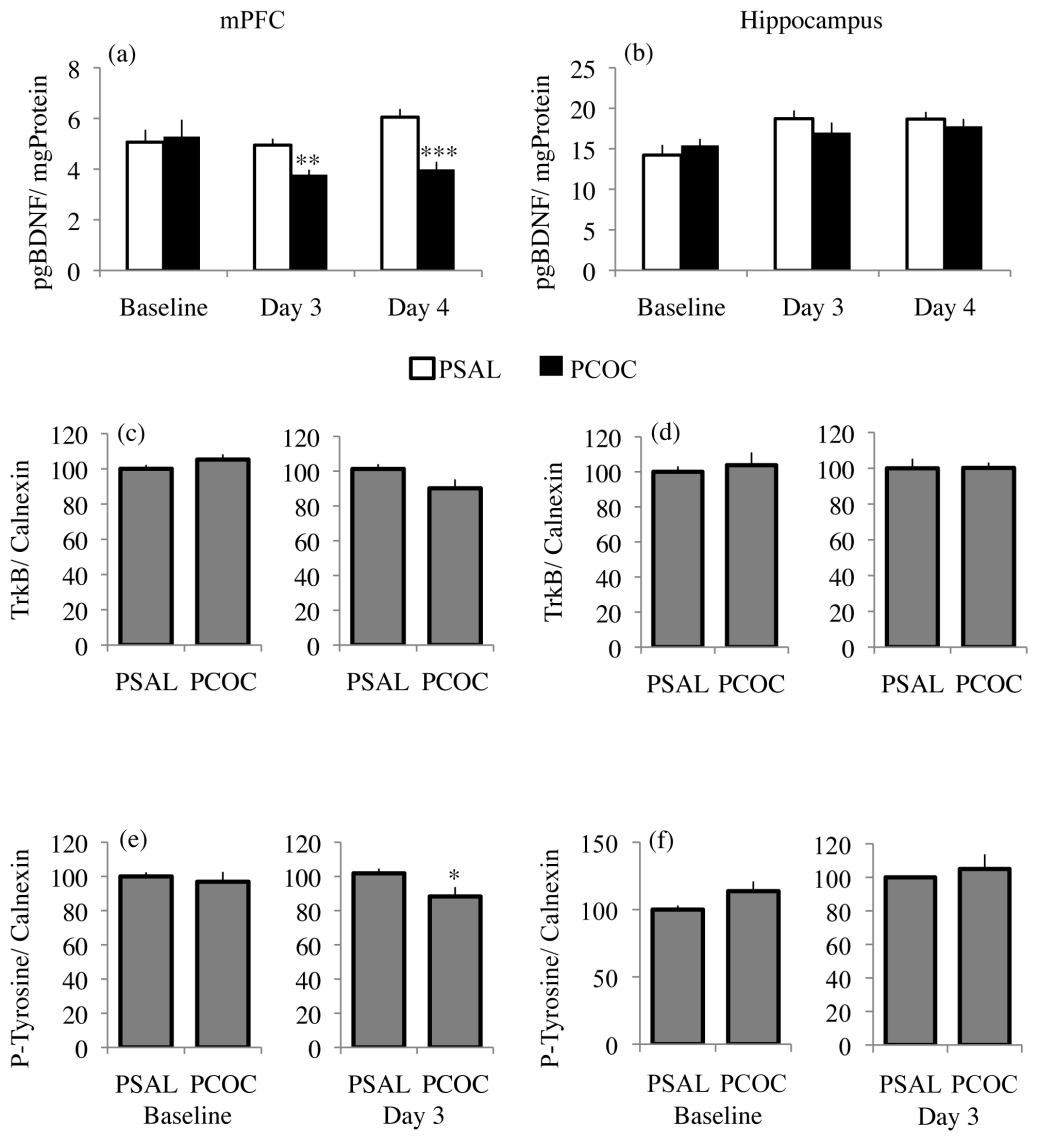
Decreased mature BDNF protein and phosphorylated TrkB protein levels in the mPFC of prenatal cocaine exposed mice during fear extinction. (**a**) In the mPFC, there was significantly decreased mBDNF protein in PCOC mice compared to PSAL mice on day 3 and day 4. (**b**) In the hippocampus, there was no change in mBDNF protein levels at baseline, day 3 or day 4. (**c**) In the mPFC of PCOC mice compared to PSAL mice there was no change in total TrkB levels at baseline. However, at day 3 there was a trend (p=0.08) towards decreased TrkB protein levels in the mPFC of PCOC mice compared to PSAL mice. (**d**) There was no change in total TrkB protein levels in the hippocampus of PCOC mice at baseline or at day 3. (**e**) In the mPFC of PCOC mice compared to PSAL mice there was no change in P-Tyrosine protein levels at baseline but significantly decreased P-Tyrosine levels on day 3. (**f**) There was no change in P-Tyrosine protein levels in the hippocampus of PCOC mice at baseline or day 3. (*p<0.05, **p<0.01, ***p<0.001 PCOC vs. PSAL). Error bars represent the mean ± SEM (mBDNF - Baseline: PSAL n=9 from 9 litters, PCOC n=11 from 11 litters; Day 3: PSAL n=8 from 8 litters, PCOC n=8 from 8 litters; Day 4: PSAL n=8 from 8 litters; PCOC n=8 from 8 litters; TrkB/ P-Tyrosine - Baseline: PSAL n=8 from 8 litters, PCOC n=8 from 8 litters; Day 3: PSAL n=9 from 9 litters, PCOC n=8 from 8 litters).

Examination of mBDNF protein levels in the mPFC revealed a main effect of prenatal treatment (F_1,47_=4.094; p=0.0487) with no effect of day or an interaction between prenatal treatment and day. While there was no difference in mBDNF protein levels in the mPFC of PCOC mice compared to PSAL mice at baseline, there was significantly decreased mBDNF protein levels on day 3 (p=0.005) and day 4 (p<0.001) of fear extinction (Figure 2a). The changes in mBDNF observed in the mPFC of PCOC mice were not evident in the hippocampus. However, in the hippocampus there was a significant effect of day, (F_2,651_=4.718; p=0.0132) though not of prenatal treatment, nor an interaction between prenatal treatment and day. Taken together, the above results indicate region-specific effects of prenatal cocaine exposure resulting in significantly decreased mBDNF protein levels in the mPFC that become evident during the consolidation phase of the extinction memory. 

To demonstrate that the decrease in mBDNF protein reported above is associated with a corresponding decrease in BDNF signaling via its TrkB receptor, levels of phosphorylated TrkB (P-Tyrosine) were measured. Calnexin, an integral protein of the endoplasmic reticulum was used to normalize for differences in protein loading as we observed no change in calnexin mRNA levels in the mPFC of adult prenatal cocaine exposed adult mice (data not shown). Additionally, we used calnexin to normalize for P-Tyrosine analyses data. In the mPFC of PCOC mice there was no change in total TrkB protein levels at baseline, though there was a trend (p=0.08) towards decreased TrkB protein levels on day 3 (Figure 2c). In the hippocampus there was no change in total TrkB protein levels at either baseline or day 3 (Figure 2d). When measuring P-Tyrosine protein levels, we found no difference between PCOC and PSAL mice at baseline in the mPFC (Figure 2e). However, on day 3 there was significantly decreased P-Tyrosine levels in the mPFC of PCOC mice compared to PSAL mice (p<0.05; Figure 2e). In the hippocampus there was no difference in P-Tyrosine levels between PCOC and PSAL mice at either baseline or on day 3 (Figure 2f). These results suggest that the lower mBDNF levels in the mPFC of PCOC mice during the consolidation phase of the extinction memory (Figure 2a), results in impaired downstream BDNF signaling through its TrkB receptor.

### Prenatal cocaine exposure results in altered mRNA levels of Bdnf splice variants in the mPFC during consolidation of the extinction memory

**Figure 3:**
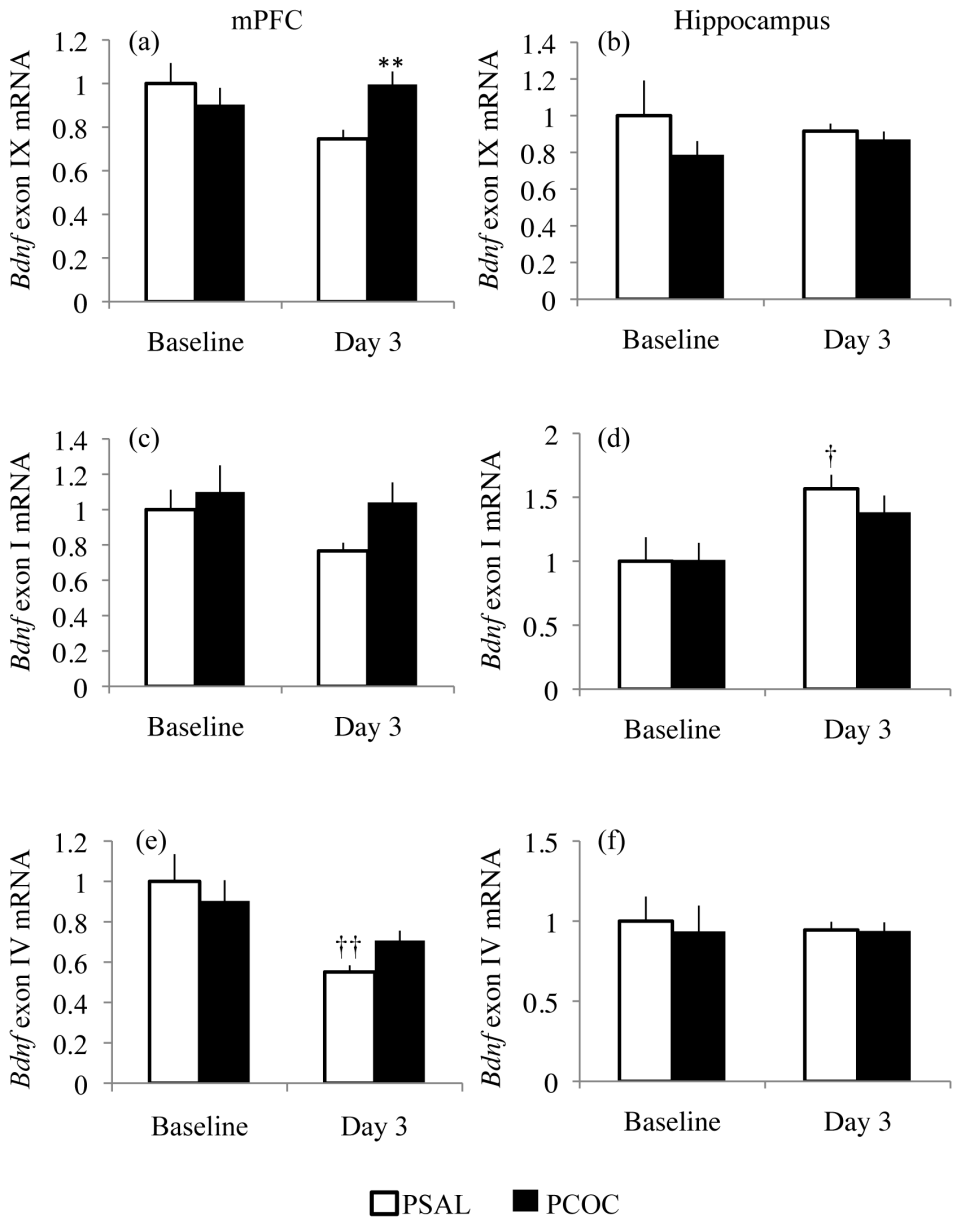
Altered *Bdnf* mRNA expression in the mPFC of prenatal cocaine exposed mice during fear extinction. (**a**) There was no change in *Bdnf* exon IX mRNA levels in the mPFC at baseline. However, at day 3 there was significantly increased BDNF exon IX mRNA levels in the mPFC of PCOC mice compared to PSAL mice. (**b**) There was no change in BDNF exon IX mRNA levels in the hippocampus at baseline or on day 3. (**c**) There was no change in BDNF exon I mRNA in the mPFC of PCOC mice at baseline or on day 3. (**d**) In the hippocampus of PSAL mice, there were higher levels of BDNF exon I mRNA levels on day 3 relative to their levels at baseline. (**e**) There was decreased BDNF exon IV mRNA levels in the mPFC of PSAL mice on day 3 compared to its levels at baseline. (**f**) There was no change in BDNF exon IV mRNA levels in the hippocampus at baseline or day 3. (*p<0.05, PCOC vs. PSAL; †p<0.05, ††p<0.01 vs. baseline of that prenatal treatment). Error bars represent the mean ± SEM (Baseline: PSAL n=12 from 12 litters, PCOC n=14 from 14 litters; Day 3: PSAL n=9 from 9 litters, PCOC n=10 from 10 litters).

Expression of the activity-driven *Bdnf* exons I and IV as well as total *Bdnf* mRNA levels (exon IX) were examined. Although we observed no change in mBDNF protein levels in the hippocampus of PCOC mice during consolidation of the extinction memory, we additionally studied *Bdnf* transcript levels in this region to further characterize the region specific effects of prenatal cocaine exposure. In the mPFC, there was an interaction between prenatal treatment and day for *Bdnf* exon IX levels (F_1,43_=5.149; p=0.0283). While we observed no change in BDNF exon IX mRNA levels at baseline, we observed significantly decreased BDNF exon IX mRNA levels in the mPFC of PSAL mice compared to PCOC mice on day 3 (p<0.01; Figure 3a). For BDNF exon I we observed no effect of prenatal treatment, day or an interaction between the two (Figure 3c). For BDNF exon IV, we observed a main effect of day (F_1,42_=9.916; p=0.003), but no effect of prenatal treatment or an interaction between the two. On day 3 we observed significantly lower levels of BDNF exon IV mRNA in the mPFC of PSAL mice (p<0.01) but not PCOC mice compared to their levels at baseline (Figure 3e). In the hippocampus there was no effect of prenatal treatment, day or an interaction between the two for *Bdnf* exons IX and IV. However, for *Bdnf* exon I there was a main effect of day (F_1,41_=9.649; p=0.0034) with significantly increased *Bdnf* exon I mRNA levels in the hippocampus of PSAL (p<0.01) mice on day 3 relative to baseline (Figure 3d). Together, these results further confirm the region specific effects of prenatal cocaine exposure as evident by a lack of dynamic regulation of *Bdnf* mRNA levels in the mPFC, but not the hippocampus during consolidation of fear extinction. 

### Prenatal cocaine exposure results in decreased binding of MeCP2 and P-CREB at Bdnf exon I and IV promoters at baseline

**Figure 4:**
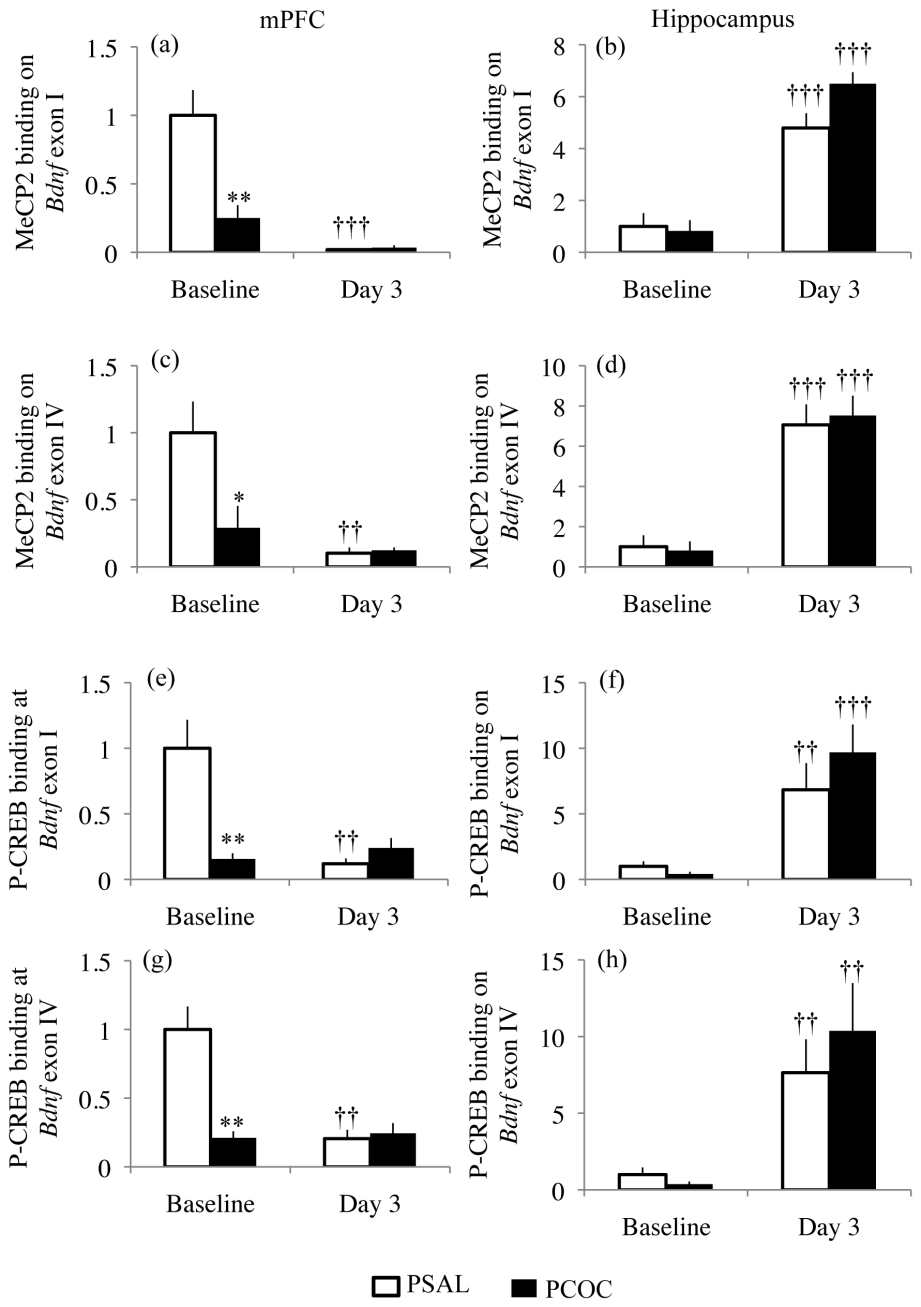
Decreased constitutive MeCP2 and P-CREB binding at *Bdnf* exon I and IV promoters in the mPFC of prenatal cocaine exposed mice. (**a**, **c**, **e**, **g**) In the mPFC of PCOC mice there was decreased binding of MeCP2 (a,c) and P-CREB (e,g) at the promoters of *Bdnf* exons I and IV at baseline. On day 3 compared to baseline, there was lower MeCP2 (a, c) and P-CREB (e, g) binding at the *Bdnf* exon I and IV promoters in PSAL mice but not PCOC mice. (**b**, **d**, **f**, **h**) In the hippocampus at day 3 compared to baseline, there were higher levels of MeCP2 and P-CREB binding at the promoter of *Bdnf* exons I and IV in PSAL and PCOC mice. (*p<0.05, **p<0.01 PCOC vs. PSAL; ††p<0.01, †††p<0.001 vs. baseline of that prenatal treatment). Error bars represent the mean ± SEM (Baseline: MeCP2: PSAL n=11 from 11 litters, PCOC n=12 from 12 litters; P-CREB: PSAL n=8 from 8 litters, PCOC n=8 from 8 litters; Day 3: MeCP2: PSAL n=14 from 14 litters, PCOC n=14 from 14 litters; P-CREB: PSAL n=14 from 14 litters, PCOC n=14 from 14 litters).

To identify aspects of such altered transcriptional regulation following prenatal cocaine exposure, the binding of MeCP2 and P-CREB were measured at the promoter regions of *Bdnf* exons I and IV. In the mPFC, for MeCP2 and P-CREB binding at the *Bdnf* exon I promoter there was a main effect of prenatal treatment (MeCP2: F_1,38_=15.47; p=0.0003; P-CREB: F_1,38_=12.54; p=0.0011), a main effect of day (MeCP2: F_1,38_=41.09; p<0.0001; P-CREB: F_1,38_=15.14; p=0.0004), and an interaction between prenatal treatment and day (MeCP2: F_1,38_=16.76; p=0.0002; P-CREB: F_1,38_=22.19; p<0.0001). For MeCP2 and P-CREB binding at *Bdnf* exon IV promoter in the mPFC there was a main effect of prenatal treatment (MeCP2: F_1,43_=8.038; p=0.007; P-CREB: F_1,37_=15.74; p=0.0003), a main effect of day (MeCP2: F_1,43_=6.756; p<0.0001; P-CREB: F_1,37_=16.25; p=0.0003), and an interaction between prenatal treatment and day (MeCP2: F_1,43_=9.056; p=0.0044; P-CREB: F_1,37_=19.13; p<0.0001). At baseline, there was significantly decreased MeCP2 (p<0.05) and P-CREB (p<0.01) binding at the promoters of *Bdnf* exons I (Figures 4a, 4e) and IV (Figures 4c, f) in the mPFC of PCOC mice compared to PSAL mice. However, at day 3 there was no difference in MeCP2 and P-CREB binding at these promoters in PCOC mice (Figure 4). Furthermore, in the mPFC of PSAL mice there was significantly lower binding of MeCP2 (p<0.01) and P-CREB (p<0.01) on the promoters of *Bdnf* exons I (Figures 4a, 4e) and IV (Figures 4c, 4g) on day 3 compared to baseline. This dynamic regulation of binding of MeCP2 and P-CREB at *Bdnf* exon I and IV promoters in the mPFC of PSAL mice evident during consolidation of fear extinction was not observed in PCOC mice. 

In the hippocampus there was a main effect of day, but not prenatal treatment or an interaction between the two for MeCP2 and P-CREB binding at the promoters of *Bdnf* exons I (MeCP2: F_1,39_=75.18; p<0.0001; P-CREB: F_1,40_=16.21; p=0.0002) and IV (MeCP2: F_1,39_=43.13; p<0.001; P-CREB: F_1,38_=11.95; p=0.0014). On day 3 compared to baseline there was higher MeCP2 (p<0.00001) and P-CREB (p<0.001) binding in PSAL and PCOC mice at the promoters of *Bdnf* exons I (Figures 4b, 4f) and IV (Figures 4d, 4h). These results once again confirm a lack of dynamic regulation of the binding of transcription factors to the *Bdnf* gene in the mPFC but not the hippocampus of PCOC mice as evident by unaltered binding of MeCP2 and P-CREB specifically within the mPFC during consolidation of fear extinction.

### Infusion of recombinant BDNF protein into the IL of prenatal cocaine exposed mice after extinction learning on day 3 rescued the behavioral deficit

**Figure 5:**
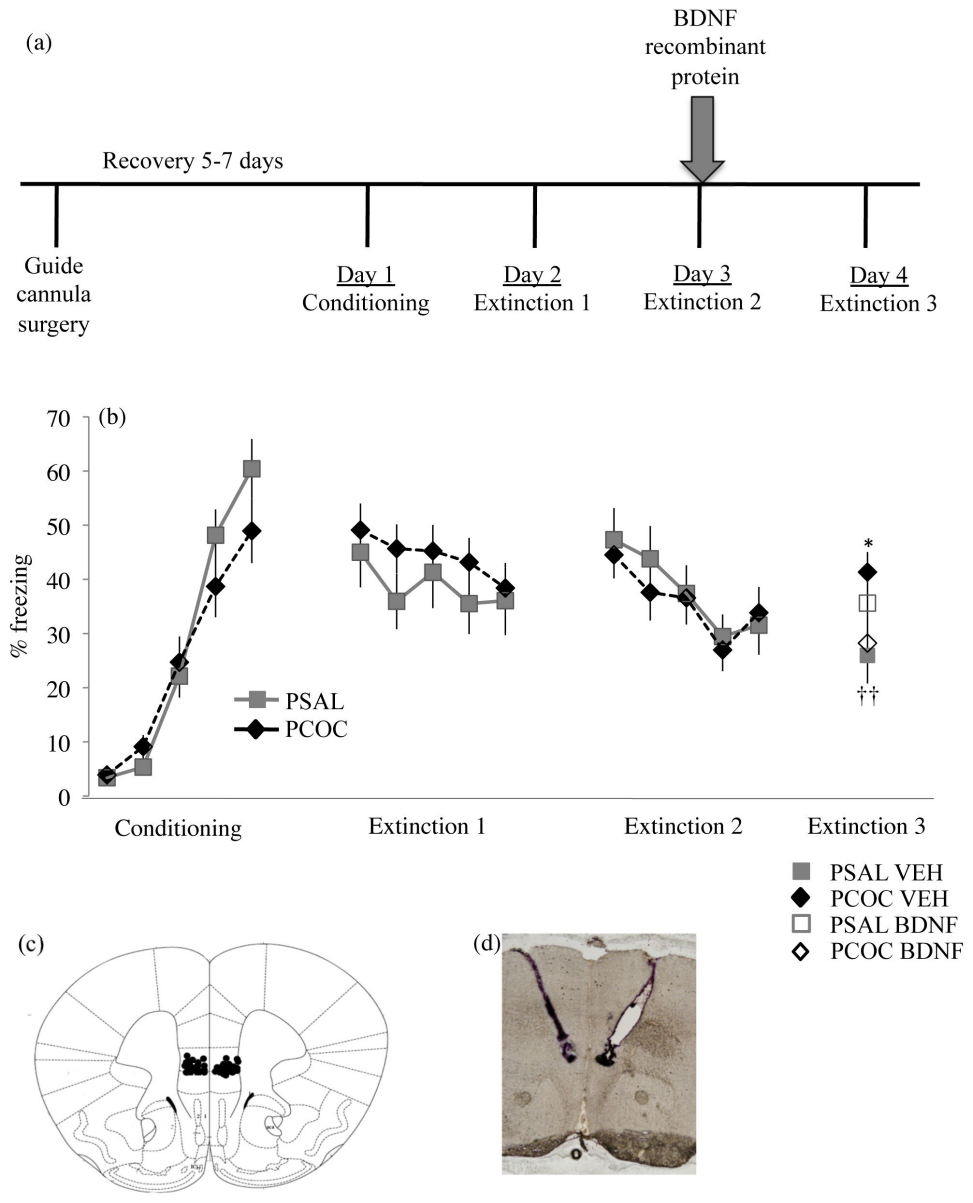
Exogenous infusion of BDNF protein into the IL of PCOC mice normalizes the deficit in recall of an extinguished cue-conditioned fear. (**a**) Overview of the experimental paradigm. Guide cannulae, targeting the infralimbic subdivision of the mPFC bilaterally were surgically implanted into the mice after which they were allowed to recover for 5-7 days before behavioral testing. Immediately after the animals went through extinction training session 2 on day 3 of the behavioral paradigm, vehicle (0.9% saline; VEH) or recombinant BDNF protein (BDNF) was infused into the infralimbic cortex and animals were tested 24 hours later for extinction recall. (**b**) Percent freezing to the tone. On day 1, both prenatal treatment groups showed significant levels of acquisition with no difference in freezing between PSAL and PCOC mice. On days 2 and 3, both prenatal treatment groups showed significant within-session extinction with no difference in freezing between PSAL and PCOC mice. Immediately after day 3, recombinant BDNF or VEH was infused into the IL bilaterally. On day 4, PCOC VEH mice showed significantly increased freezing compared to PSAL VEH mice demonstrating a deficit in extinction recall (*p<0.05). However, PCOC BDNF mice showed significantly decreased freezing compared to PCOC VEH mice indicating a rescue of the behavioral deficit (††p<0.01). Error bars represent the mean ± SEM (PSAL VEH n=11 from 11 litters, BDNF n=9 from 9 litters; PCOC VEH n=9 from 9 litters; BDNF n=11 from 11 litters). **c**) Schematic representation of the guide cannula placements in animals that were used for behavioral testing. (**d**) A representative image of guide cannulae targeting the IL bilaterally.

To further explore whether lower mBDNF protein in the mPFC of PCOC mice on day 3 (Figure 2a) contributes to the deficit in recall of fear extinction (Figure 1a) observed in these animals on day 4, recombinant BDNF protein was infused into the IL subdivision of the mPFC immediately after day 3 of the behavior (outlined in Figure 5a). On day 1 of the behavior, PCOC and PSAL animals showed an increase in freezing with each tone-shock pairing (F_4,175_=54.88, p<0.0001) with no effect of prenatal treatment. On day 2 there was no main effect of tone or prenatal treatment. On day 3, all animals exhibited a decrease in freezing from the first to the fifth tone (main effect of tone, F_4,175_=3.668, p=0.007) with no effect of prenatal treatment suggesting no impact of prenatal cocaine exposure on extinction learning. Immediately after the animals were retrieved from the behavioral apparatus on day 3, recombinant BDNF protein (BDNF) or PBS (VEH) was infused bilaterally into the IL. On day 4 there was a significant interaction between prenatal treatment and BDNF infusion on freezing (F_1,33_=6.489, p=0.016). Post-hoc analyses revealed significantly increased freezing in PCOC VEH animals compared to PSAL VEH animals (p<0.01; Figure 5b) confirming the deficit previously observed in PCOC mice (Figure 1a). Furthermore, post-hoc analyses revealed significantly decreased freezing in PCOC BDNF animals compared to PCOC VEH animals (p<0.01; Figure 5b). These results suggest that the decreased BDNF protein evident in the mPFC of PCOC compared to PSAL mice during consolidation of the extinction memory (Figure 2a) mediated the behavioral deficit in extinction recall. 

## Discussion

In this study we observed a deficit in recall of fear extinction in adult prenatal cocaine exposed mice attributable to a decrease in mBDNF signaling through its TrkB receptor, specifically in the mPFC during consolidation of the extinction memory. This deficit was rescued by infusing exogenous recombinant BDNF protein into the IL after extinction learning. In contrast to the decreased levels of mBDNF protein, we observed a significant increase in *Bdnf* exon IX mRNA levels in the mPFC of PCOC mice during the consolidation phase of the extinction memory. Furthermore, while the control animals showed a decrease in *Bdnf* exon IV expression in the mPFC after fear extinction compared to their levels at baseline, the PCOC mice showed no change in their expression. Of particular interest are the findings at the level of the *Bdnf* gene where we observed a significant decrease in the constitutive binding of the transcription factors MeCP2 and P-CREB at the *Bdnf* exon I and IV promoters in the mPFC of PCOC mice that unlike PSAL mice remained unchanged by extinction learning. Contrary to our findings in the mPFC, in the hippocampus we did not observe prenatal cocaine-induced changes in *Bdnf* mRNA, the binding of transcription factors MeCP2 and P-CREB to the *Bdnf* gene, or mBDNF protein expression either at baseline or during the consolidation phase of the extinction memory. These findings provide support for long-lasting cognitive deficits in PCOC mice as a result of a lack of learning-induced molecular changes specifically within the mPFC of these animals.

In our behavioral paradigm, the control animals displayed extinction of a cue-conditioned fear by day 4 of the behavioral paradigm while the prenatal cocaine exposed mice continued to display enhanced freezing to the tone despite normal within-session extinction learning. These results suggest that prenatal cocaine exposure does not impact the acquisition of the extinction memory but impairs consolidation of the extinction memory. Since we observed no differences in the conditioned response between the prenatal groups during the acquisition, consolidation and retrieval of the fear memory, all of which heavily recruit the amygdala [41], we hypothesize that prenatal cocaine exposure does not affect amygdala function and associative learning. These results are in agreement with our previous findings where we see no effect of prenatal cocaine exposure on fear acquisition, consolidation and retrieval in mice that were heterozygous for the *Bdnf* Val66Met allele [10]. This suggests that molecular maladaptations in the mPFC of prenatal cocaine exposed mice may be responsible for the behavioral deficit in fear extinction that we have identified. Our findings may also partially reflect a more general impairment in frontal lobe function such as a prenatal cocaine-induced deficit in second-order conditioning (i.e., blocking), a finding that has been reported previously in prenatal cocaine exposed animals [7,42].

Our behavioral findings raise the possibility that prenatal cocaine exposure increases the risk of developing neuropsychiatric symptoms associated with learning of fear associated cues such as is seen in post-traumatic stress disorder (PTSD). A similar hypothesis has been suggested for individuals exposed to prenatal nicotine [34]. Furthermore, since there is evidence showing increased prevalence of PTSD among pregnant women who abuse cocaine [35], it is possible that such problematic behaviors may result from an interaction between direct effects of cocaine exposure and genetic factors [36], potentially including naturally occurring polymorphisms for the BDNF allele in humans. A meta-analysis of longitudinal clinical studies have identified a significant independent role of prenatal cocaine exposure in contributing to externalizing behavioral problems [37] as subsequently confirmed by associations with increased [38] and earlier [39] use of drugs of abuse in exposed offspring as teenagers. The origins of such risky behaviors may be attributable to the emergence of neurobehavioral disinhibition evident in adolescents as a result of prenatal cocaine exposure [36,40]. Such clinical data is consonant with the findings we report in our preclinical prenatal cocaine model, emphasizing the role that prenatal drug exposure may confer in altering the maturation and integrity of frontal cortical function, imparting greater vulnerability to PTSD in exposed offspring. 

By infusing recombinant BDNF protein into the infralimbic cortex of the mPFC of prenatal cocaine exposed mice immediately after extinction learning, we were able to rescue the deficit in extinction of a cue-conditioned fear in these animals indicating that the behavioral deficit was a result of decreased BDNF protein in this region. However, surprisingly in the saline treated control animals BDNF infusion did not enhance their extinction as has been previously reported by others doing similar studies in rats [19]. This could be due to the difference in species, and/or an increased spread of the BDNF protein encroaching into the more dorsal prelimbic cortex, a region that has been shown to regulate the fear response [43].

At the molecular level we found no effect of prenatal cocaine exposure on the constitutive expression of *Bdnf* mRNA and protein in the mPFC and hippocampus. Surprisingly, at the level of the *Bdnf* gene we observed a marked decrease in the constitutive binding of MeCP2 and P-CREB at the promoters of *Bdnf* exons I and IV specifically in the mPFC of PCOC mice. We hypothesize that the decreased constitutive binding of MeCP2 and P-CREB at these *Bdnf* promoters may prime the animal to react differently when subjected to an external stimulus (i.e. learning) due to an alteration in stimulus-induced changes in *Bdnf* transcription. Further experiments utilizing transgenic mice that have specific mutations at the sites at which MeCP2 and P-CREB bind the *Bdnf* exon I and IV promoters (as used in [44]) will need to be conducted to confirm the relevance of changes in the binding of these transcription factors on stimulus-induced *Bdnf* expression during extinction of fear learning. 

The mismatch between decreased BDNF protein and increased induction of *Bdnf* mRNA that we observed in the mPFC of PCOC mice has previously been shown in the prefrontal cortex (PFC) after repeated cocaine exposure [45]. Furthermore, the mature BDNF protein being measured in the mPFC may not necessarily be endogenously produced in this anatomical region; BDNF in the PFC has been shown in part, to take origin in other cortical and subcortical regions [46]. Additionally, the increased *Bdnf* mRNA evident in the mPFC of PCOC mice may result in increased BDNF protein expression in other brain areas innervated by the mPFC. This suggests that alterations in BDNF signaling in other brain regions impacted by mPFC activity may additionally mediate aspects of the behavioral deficit evident in PCOC mice. 

Of note, the decrease in *Bdnf* exon IV mRNA levels in the mPFC of control animals after fear extinction is in contrast to what has been previously published by others [47]. This discrepancy could be due to the anatomical region being studied (PFC in Bredy et al., 2007 vs. the more discrete mPFC that we studied) as well as the behavioral protocol being employed. It has been shown that membrane depolarization induced transcriptional and translational initiation of *Bdnf* requires a minimum of 60 -180 minutes [48]. In our study the behavioral paradigm employed lasted a total of 7.5 mins and tissue was collected immediately after the behavior. Therefore, the altered mRNA levels of *Bdnf* observed in the mPFC of prenatally cocaine exposed mice is reflective of lower *Bdnf* levels present prior to the behavioral testing rather than a result of the behavior itself. In contrast, in Bredy et al. (2007) the animals were sacrificed two hours after the behavior and hence the changes reported would be a reflection of the extinction session itself.

To conclude, our study is the first to identify a molecular basis, namely decreased BDNF protein in a particular neuroanatomical region, specifically mPFC as the determinant of a discrete behavioral deficit in PCOC animals. Furthermore, this study has identified prenatal cocaine-induced constitutive modifications in the binding of transcriptional regulators at the *Bdnf* gene that show a lack of inducibility with learning. Understanding such molecular and behavioral alterations in PCOC mice is essential for designing improved therapeutic interventions for the prevention and treatment of cognitive and behavioral disorders observed in this population.
